# GRK3 as a Prognosis Biomarker in Gastric Cancer

**DOI:** 10.7150/jca.64748

**Published:** 2022-01-31

**Authors:** Chia-Lang Fang, Yu-Feng Tian, Shiau-Shiuan Lin, Shih-Ting Hung, You-Cheng Hseu, Chun-Chao Chang, Chia-Lin Chou, Li-Chin Chen, Wen-Ching Wang, Kai-Yuan Lin, Ding-Ping Sun

**Affiliations:** 1 Department of Pathology, School of Medicine, College of Medicine, Taipei Medical University, Taipei, Taiwan.; 2 Department of Pathology, Taipei Medical University Hospital, Taipei Medical University, Taipei, Taiwan.; 3 Department of Surgery, Chi Mei Medical Center, Tainan, Taiwan.; 4 National Tainan Second Senior High School, Tainan, Taiwan.; 5 Department of Medical Research, Chi Mei Medical Center, Tainan, Taiwan.; 6 Department of Cosmeceutics, China Medical University, Taichung, Taiwan.; 7 Department of Health and Nutrition Biotechnology, Asia University, Taichung, Taiwan.; 8 Chinese Medicine Research Center, China Medical University, Taichung, Taiwan.; 9 Department of Internal Medicine, Taipei Medical University Hospital, Taipei Medical University, Taipei, Taiwan.; 10 Department of Internal Medicine, School of Medicine, College of Medicine, Taipei Medical University, Taipei, Taiwan.; 11 Department of Biotechnology, Chia Nan University of Pharmacy and Science, Tainan, Taiwan.; 12 Department of Food Science and Technology, Chia Nan University of Pharmacy and Science, Tainan, Taiwan.

**Keywords:** GRK3, Gastric cancer, Prognosis, Biomarker

## Abstract

**Background:** Globally, gastric cancer is ranked 4th and 3rd in terms of incidence and mortality rate among all cancer types. This study aimed to examine the relationship between G protein-coupled receptor kinase 3 (GRK3) and gastric cancer prognosis and investigate the role of GRK3 in gastric cancer carcinogenesis.

**Methods:** GRK3 level in gastric tissues and cells were determined using immunohistochemistry and immunoblotting. Kaplan-Meier analysis with the log-rank test was employed to evaluate the relationship between GRK3 expression and gastric cancer prognosis. RNAi technology was applied to examine the effects of GRK3 inhibition on gastric cancer proliferation and spread.

**Results:** GRK3 overexpression was correlated significantly with lymphatic metastasis (P = 0.0011), distant metastasis (P < 0.0001), TNM stage (P = 0.0035), and vascular invasion (P = 0.0025). Kaplan-Meier survival analysis showed that the disease-free survival and overall survival of patients with high GRK3 expression were significantly shorter than those of patients with low GRK3 expression. Multivariate Cox regression analysis also showed that the overexpression of GRK3 was an independent prognostic biomarker of gastric cancer (P = 0.029). In cultured gastric cancer cells, GRK3 knockdown inhibited cell proliferation, migration, and invasion. Further analysis revealed that more GRK3-knockdown cells were in G0/G1 phase and few cells were in S phase, thereby inhibiting cell proliferation.

**Conclusions:** GRK3 overexpression can be a candidate biomarker for gastric cancer prognosis. GRK3 is also a potential therapeutic target for gastric cancer.

## Introduction

Gastric cancer (GC) is ranked 3^rd^ in terms of mortality rate among all cancer types 1. Although the incidence of GC is decreasing, the prognosis of patients with GC remains poor. A 2019 government report indicated that approximately 3,600 people were diagnosed with GC and GC caused over 2,000 deaths in 2106 in Taiwan. Clinical treatment of patients with GC remains challenging owing to the lack of understanding of the pathogenesis of GC and the lack of targeted gene therapies 2, 3. Nevertheless, advances in molecular biology techniques have provided possibilities for exploring GC-related factors, including oncogenes and tumor suppressor genes, which can be used as novel biomarkers for GC 4. Some aberrantly expressed molecules such as USP3, TMPO-α, NCAPG, KLF16, and RAD18 could be used as prognostic biomarkers for GC 5-9.

The G protein-coupled receptor kinase (GRK) family includes seven serine/threonine kinases. The main function of these proteins is to phosphorylate G protein-coupled receptors to inhibit their function 10-12. The expression of G protein-coupled receptor kinase 3 (GRK3) has been reported in various cancers. Billard et al. analyzed data from the TGCA database and found that GRK3 expression in breast cancer tissues was lower than that in normal tissues 13. Jin et al. employed immunoblotting and immunohistochemistry to measure GRK3 expression in liver cancer tissues and found that GRK3 expression level in liver cancer tissues was lower than that in normal tissues 14. In contrast, GRK3 expression is significantly increased in other cancers. Jiang et al. employed real-time quantitative polymerase chain reaction and immunohistochemistry to measure the expressions of GRK3 mRNA and protein in colon cancer tissues. It was found that GRK3 mRNA and protein expressions in colon cancer tissues were higher than those in normal tissues 15. Similarly, Liu et al. employed immunohistochemistry and found that GRK3 protein expression level in pancreatic cancer tissues was higher than that in normal tissues 16. In summary, the expression level of GRK3 in different cancers suggests that it may act as an oncogene or a tumor suppressor gene in different cancers, depending on the tissue type, cancer type, and cancer stage. At present, the expression level of GRK3 in GC remains unknown.

As G protein-coupled receptor-related signal transduction is extremely important for tumor growth and metastasis, understanding how GRKs regulate G protein-coupled receptor activity in cancer cells will promote our understanding of tumorigenesis and oncogenes and help develop new cancer treatment methods and drugs. Billard et al. were the first to examine the role of GRK3 in the occurrence of breast cancer. GRK3 knockdown in MDA-MB-231 and MDA-MB-468 cells inhibits CXCL-12-mediated chemotaxis. This demonstrates that GRK3 can regulate CXCR4-mediated CXCL-12 activation 13. More, stable GRK3 knockdown facilitates metastasis of xenografted breast cancer cells.

At present, only few studies have discussed the correlation between GRK3 and cancer prognosis, and their conclusions are inconsistent. Jin et al. found that low GRK3 expression in liver cancer tissues is positively correlated with poor patient prognosis 14. In contrast, Jiang et al. found that GRK3 protein overexpression in colon cancer tissues is positively correlated with poorer patient prognosis 15. Hence, more studies are required to clarify the correlation between GRK3 and cancer prognosis. Up until now, the correlation between GRK3 and GC prognosis remains unknown.

In this study, we examined the expression of GRK3 in GC and assessed the correlation between GRK3 expression and clinicopathologic characteristics of GC and patient survival. We also examined the role of GRK3 in regulating cell proliferation, migration, and invasion.

## Materials and methods

### Patients and tissue specimens

For this study, the paired tumor and adjacent normal samples were collected from 144 GC patients at Taipei Medical University Wan Fang Hospital between 1998 and 2011. None of these patients had received preoperative chemotherapy and/or radiotherapy. This study was performed in accordance with the Helsinki declaration and was approved by the Research Ethics Committee of Taipei Medical University Wan Fang Hospital (Approval No. 99049). Informed consent was obtained from all the participants.

### Cell culture

The human normal gastric cell line Hs738.St/Int (provided by the American Type Culture Collection; Manassas, VA, USA) was cultured in DMEM. GC cell line AGS (provided by the Bioresource Collection and Research Center; Hsinchu, Taiwan) was grown in F-12K. GC cell lines, including NCI-N87, TMC-1, TSGH 9201 (provided by the Bioresource Collection and Research Center; Hsinchu, Taiwan), SK-GT-2 (provided by the European Collection of Cell Cultures; Salisbury, UK), and 23132/87 (provided by Creative Bioarray; Shirley, NY, USA), were maintained in RPMI-1640. GC cell line HGC-27 (provided by the European Collection of Cell Cultures; Salisbury, UK) was cultured in MEM. All media were supplemented with 10% fetal bovine serum and antibiotics. All cell lines were authenticated by cell providers. We also checked the ICLAC database to ensure that all cell lines were not misidentified.

### Immunohistochemistry

**Figure 1:**
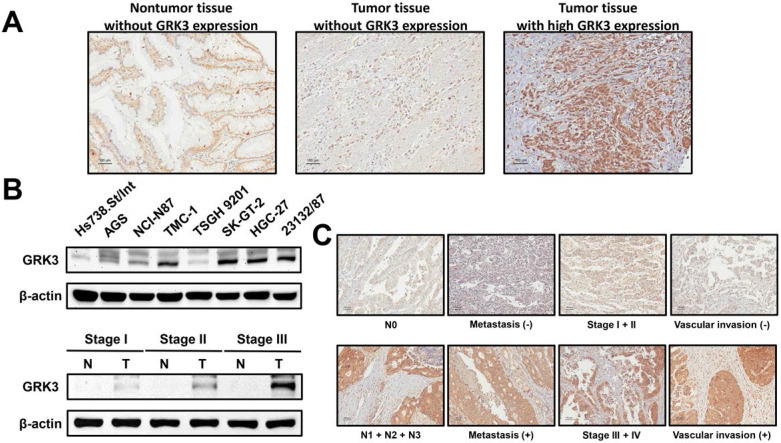
** GRK3 expression in gastric tissues and cell lines. (A)** GC analyzed by immunostaining with an antibody against GRK3. Left panel shows a nontumor sample without GRK3 expression; middle panel shows a tumor sample without GRK3 expression; right panel shows a tumor sample with high GRK3 expression. Magnification: 200×. **(B)** Endogenous GRK3 protein expression was remarkably increased in GC cell lines and tissues. (C) The representative GRK3 staining for different clinicopathologic characteristics. Magnification: 200×.

The immunoreactivity was detected using the conventional peroxidase-conjugated streptavidin-biotin method (Dako REAL EnVision Detection System; Dako, Carpinteria, CA, USA). The primary antibody against GRK3 (purchased from LifeSpan, Cat. No. LS-C164294, Seattle, WA, USA) were added to the paraffin-embedded sections overnight at 4°C. Figure S1 showed the whole blot of GRK Western blotting in gastric tissues. In this blot, GKR3 was the major band. It was thought that the immunostaining shown in Figure 1A was mostly contributed by the major GRK3 band. Human hepatocellular carcinoma was previously demonstrated to be positive for GRK3. Negative controls were included by substituting the primary antibody with 1× phosphate buffer saline (Corning). Photos were captured with BX51 microscope (Olympus, Tokyo, Japan). The staining intensity of GRK3 was defined as follows: 0, no staining; 1, weak and focal staining in < 25% of the tissue; 2, moderate staining in 25%-50% of the tissue; and 3, strong staining in > 50% of the tissue. Patients with a score of 0 or 1 of GRK3 expression were designated negative for GRK3, and patients with a score of 2 or 3 were designated positive for GRK3. All stained sections were scored by an experienced pathologist (Fang CL) whthout prior knowledge of the clinicopathologic parameters and clinical outcomes of the patients.

### Protein isolation and Western blotting

Isolation of total proteins from cells and tissues were performed using RIPA Buffer (Thermo). Protein samples were denatured and separated using 10% SDS-PAGE. After electrophoresis, the proteins were transferred to nitrocellulose membranes and the blocked membranes were added with GRK3 antibody overnight at 4 °C. β-Actin was used as a loading control. After incubation with peroxidase-conjugated secondary antibodies (Sigma), enhanced chemiluminescence reagents (Thermo) were employed to visualize the protein signals. GeneTools software (Syngene, Cambridge, UK) was used to process the images.

### shRNA treatment

Lentiviral vectors (two GRK3-shRNA constructs, clone IDs: TRCN0000002036, TRCN0000320947, and one control, clone ID: pLKO_TRC025) were purchased from the National RNAi Core Facility, Taipei, Taiwan. For shRNA treatment, HGC-27 and AGS cells were infected with lentiviral vectors and stable clones resistant to puromycin (Thermo) were selected. The effects of shRNA treatment were evaluated using Western blotting.

### Colony formation assay

Cells were seeded into 6-well plates (500 cells/well) and cultured for 12 days. Individual colonies were fixed with 10% formalin and stained with 1% crystal violet. The plates were scanned with Scanjet 2200c scanner (HP, Palo Alto, CA, USA). After scanning, methanol was added at room temperature to solubilize the dye. The optical density (OD540) was read to quantify the number of colony formed. The assay was conducted three 3 times, and the results were presented as the mean ± SD.

### Cell cycle analysis

The distribution of cell cycle phases was determined by analysis of propidium iodide-labeled cells, as described in our previous study 17.

### Wound-healing assay

Cells (5 × 10^5^) were seeded into 12-well plates and grown to 100% confluence. A wounded area was created by scratching the confluent cell monolayer with a 200 μL pipette tip. The shed cells were washed with 1× phosphate buffer saline, and the cells were then cultured for 18 hours. The migration of cells to the wounded area was monitored at 0 and 18 hours, and the wounded area was photographed (100× magnification, with Leica DMIRB microscope, Leica, Wetzlar, Germany). The number of migrated cells was quantified using Image J software. The percentage of cell migration was calculated by defining the number of migrated wild type cells as 100%. All experiments were performed in triplicate, and the results were presented as the mean ± SD.

### Cell invasion assay

The cell invasive capability was examined using a Cell Invasion Assay Kit (Merck Millipore, Darmstadt, Germany), following the manufacturer's instructions. The cells (2 × 10^5^) in serum-free media were seeded to ECMatrix-layered cell culture inserts (containing 8 μm pore size polycarbonate membranes) and complete media were added to 24-well plates. After 24 hours, the cells on the upper surface were removed, and the invaded cells on the lower surface of the membranes were stained with the Staining Solution. The photos were taken (100× magnification, with Leica DMIRB microscope), and the number of invaded cells was quantified. The percentage of cell invasion was calculated by defining the number of invaded wild type cells as 100%. The assay was conducted 3 times independently, and the results were presented as the mean ± SD.

### Statistical analysis

The χ^2^ test was performed to analyze the correlation between GRK3 level and various clinicopathologic features. Kaplan-Meier method was used to create survival curves based on high and low GRK3 immunohistochemical scores and log-rank test was used to compare disease-free and overall survival. Parameters that emerged as significant (P < 0.05) in the univariate analysis were entered as variables in the multivariate Cox regression model, and the hazard ratio (HR) and independence of prognostic impact were determined in a stepwise backward fashion. All data were analyzed using SPSS version 24.0 (IBM, Armonk, NY, USA). The differences in cell growth, migration, and invasion between control and GRK3-manipulated cells were examined using Student's t tests. All statistical tests were 2-sided, and P < 0.05 was considered significant.

## Results

### Increased GRK3 expression in GC

To understand the possible role of GRK3 in the occurrence and progression of GC, we measured GRK3 expression level in GC tissues from 144 patients. Immunohistochemistry revealed that GRK3 expression was higher in tumor tissues than in nontumor tissues (Figure 1A). Specifically, GRK3 was not expressed at all in tumor tissues from 8% of patients (interpreted score of 0), and tumor tissues from 44% of patients showed weak and localized GRK3 expression (interpreted score of 1). Tumor tissues from 47% of patients showed higher expression or overexpression of GRK3 (interpreted score of 2 in 40% of patients and interpreted score of 3 in 7% of patients). We next examined GRK3 expression in eight gastric cell lines to verify the above immunohistochemistry results. Immunoblotting revealed that GRK3 expression level was significantly increased in all GC cell lines compared with a normal gastric cell line (Figure 1B). In addition, immunoblotting also showed that GRK3 expression was higher in tumor tissues than in nontumor tissues (Figure 1B). These results strongly indicated that GRK3 is significantly increased in GC, particularly so in advanced GC.

### Correlation of increased GRK3 expression with clinicopathologic characteristics of GC and survival in patients with GC

The result of increased GRK3 expression in GC encouraged us to further understand the clinical correlation between GRK3 and GC. GRK3 expression level was significantly correlated with lymphatic metastasis, distant metastasis, TNM stage, and vascular invasion (Table 1). The significant positive correlation between GRK3 overexpression and stage was consistent with the results of immunoblotting presented in Figure 1B. Figure 1C shows the representative GRK3 staining for aforementioned clinicopathologic characteristics also indicating a correlation between GRK3 and these clinicopathologic characteristics.

**Figure 2:**
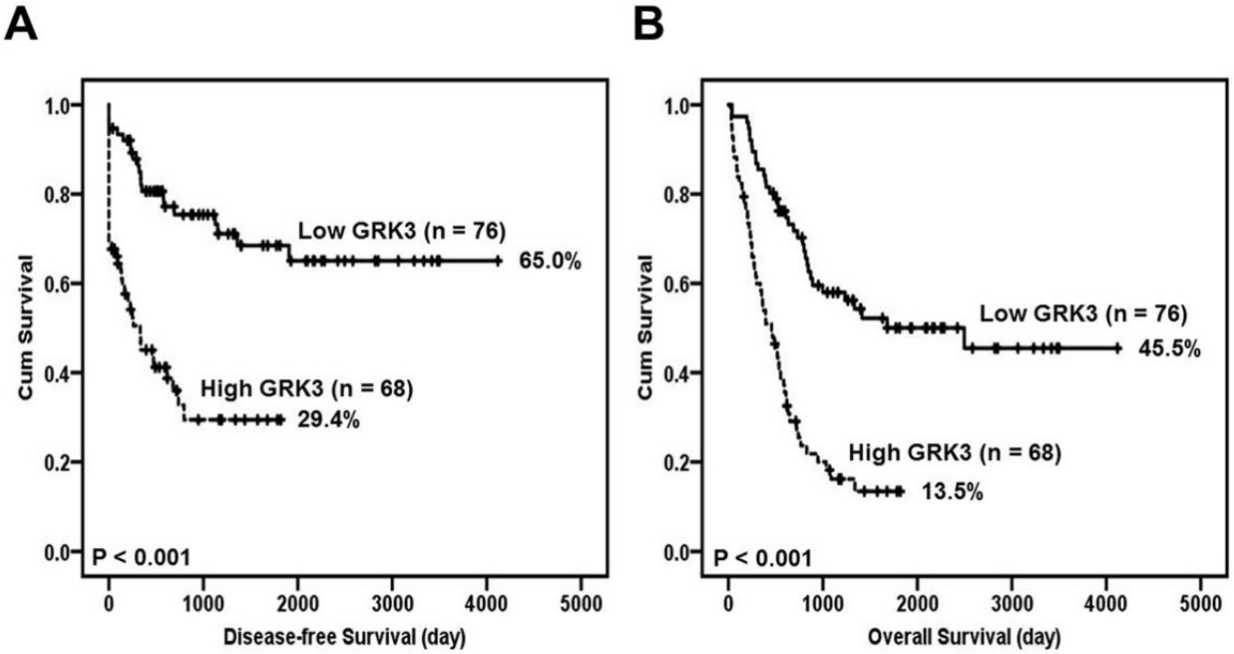
** Survival analysis of GC patients stratified by GRK3 immunoreactivity.** Panel **(A)** shows the disease-free survival. Panel **(B)** shows the overall survival. All statistical tests were 2-sided. Significance level: *P* < 0.05.

Furthermore, Kaplan-Meier method and log-rank test revealed that GC patients with high GRK3 expression had a significantly shorter disease-free survival time than the patients with low GRK3 expression (Figure 2A). The disease-free survival rate for GC patients with low GRK3 level was 0.650 (95% confidence interval [CI] 0.519-0.781). The disease-free survival rate for GC patients with high GRK3 level was 0.294 (95% CI 0.161-0.427).

Poor overall survival was significantly positively correlated with GRK3 overexpression (Figure 2B). The overall survival rate for GC patients with low GRK3 level was 0.455 (95% CI 0.314-0.596). The overall survival rate for GC patients with high GRK3 level was 0.135 (95% CI 0.043-0.227).

Table 2 summarizes the univariate analysis of the prognostic biomarkers and patient survival. GRK3 overexpression, depth of tumor invasion, lymphatic metastasis, distant metastasis, TNM stage, degree of differentiation, and vascular invasion were significantly correlated with disease-free survival.

In the multivariate analysis, only GRK3 overexpression and distant metastasis were prognostically independent (Table 2).

In summary, GRK3 overexpression seemed to be an independent predictor of poor patient prognosis. Increased GRK3 expression may promote GC progression and can be used as a biomarker for GC.

### GRK3 interference inhibits GC cell proliferation

**Figure 3:**
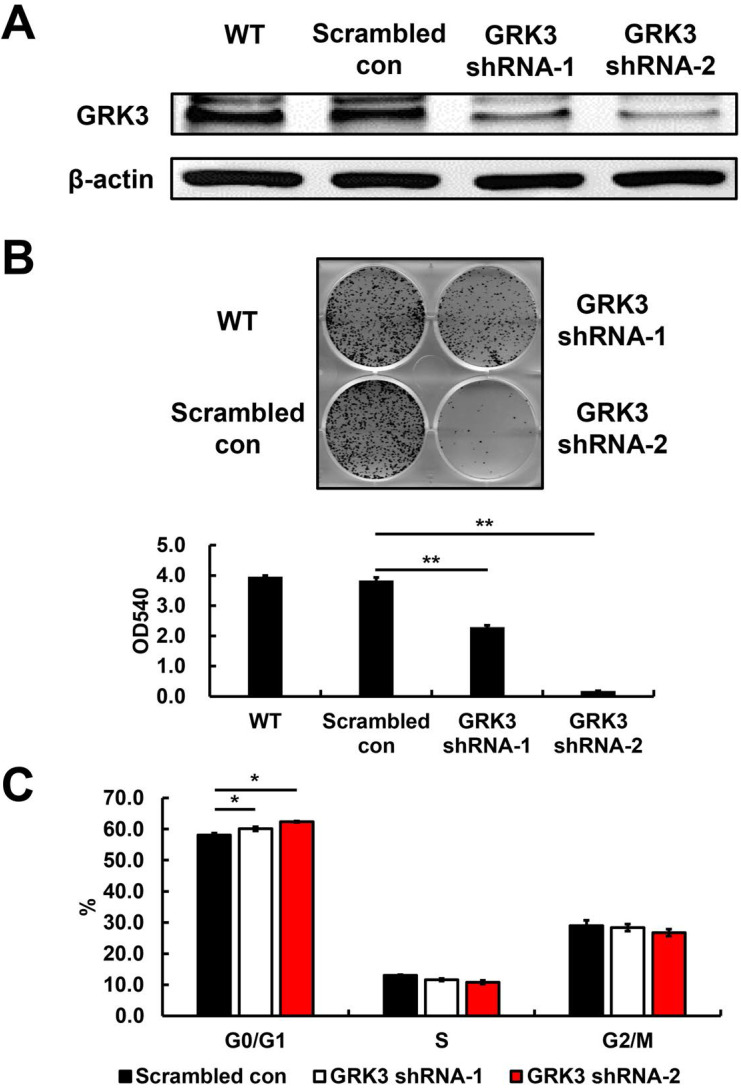
** Verification of GRK3 knockdown in HGC-27 cells, and the effect of stable GRK3 knockdown on cell growth and cell cycle distribution.** The Western blotting results **(A)** indicate GRK3 was efficiently knockdown by shRNA treatment. **(B)** Stable GRK3 knockdown resulted in remarkedly decreased colony formation. **(C)** Stable GRK3 knockdown resulted in a sustained accumulation of cells in the G0/G1 phase. Cellular distribution (as percentages) in different phases of the cell cycle (G0/G1, S, and G2/M) is presented. A typical result from three independent experiments is shown. *, P < 0.05; **, P < 0.01. WT: non-transduced HGC-27 cells; Scrambled con: scrambled control HGC-27 cells; GRK3 shRNA: GRK3-knockdown HGC-27 cells.

Based on the GRK3 expression levels in cells, we used HGC-27, a GC cell line with high GRK3 expression, to elucidate the role of endogenous GRK3 in regulating cell proliferation. We used adenoviruses carrying an shRNA vector for GRK3 to infect HGC-27 cells and obtained GRK3-knockdown HGC-27 cells (Figure 3A). As shown in Figure 3B, the colony-forming ability of GRK3-knockdown HGC-27 cells was inhibited compared with the control group. This result showed that GRK3 knockdown inhibited HGC-27 cell proliferation.

GRK3 knockdown was also conducted in AGS cells and GRK3-knockdown AGS cells were obtained (Figure S2). As HGC-27 cells, the colony-forming ability of GRK3-knockdown AGS cells was inhibited compared with the control group (Figure S2).

To understand the biological events involved in the inhibition of proliferation resulting from GRK3 knockdown, flow cytometry was used to analyze the distribution of various cell cycle phases. The percentage of G0/G1 cells was significantly increased in GRK3-knockdown HGC-27 cells (Figure 3C). Therefore, the shRNA experiment revealed that GRK3 knockdown interfered with the transition from G0/G1 phase to S phase during cell cycle, thereby inhibiting HGC-27 cell proliferation.

### GRK3 inhibition decreases spread of GC cells

**Figure 4:**
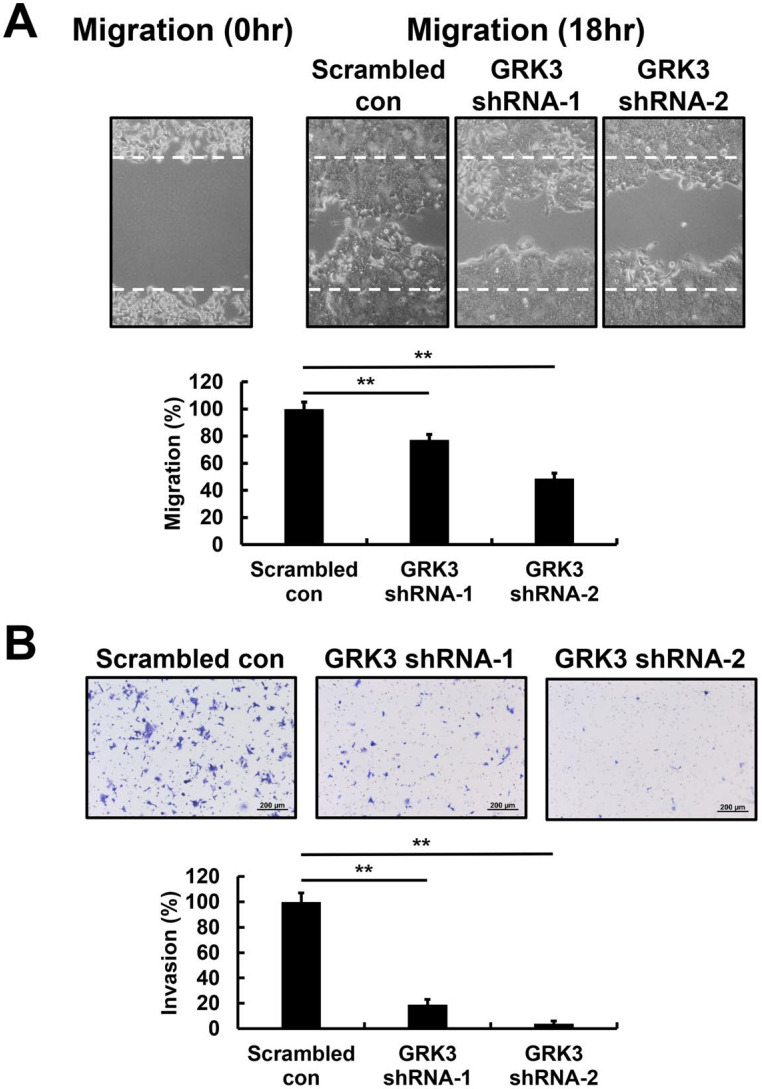
** Effect of GRK3 knockdown in HGC-27 cells on cell migration and invasion. (A)** Stable GRK3 knockdown markedly decreased cell migration. **(B)** Stable GRK3 knockdown markedly decreased cell invasion. A typical result from three independent experiments is shown. **, *P* < 0.01. WT: non-transduced HGC-27 cells; Scrambled con: scrambled control HGC-27 cells; GRK3 shRNA: GRK3-knockdown HGC-27 cells.

The wound-healing assay was employed to examine the effects of GRK3 knockdown on migration of GC cells. Wound-healing speed was significantly lower in GRK3-knockdown HGC-27 cells compared with cells in the control group (Figure 4A). Finally, in cell invasion experiments, the cell invasion speed of GRK3-knockdown HGC-27 cells was significantly inhibited compared with cells in the control group (Figure 4B). Therefore, the shRNA experiment revealed that GRK3 knockdown inhibits HGC-27 cell migration and invasion.

As HGC-27 cells, the cell migration and invasion of GRK3-knockdown AGS cells were inhibited compared with the control group (Figure S2).

## Discussion

According to statistical data from the World Health Organization Report on Cancer, over 1 million new cases of GC are diagnosed annually around the world and the proportion of male patients with GC is twice that of female patients. Hence, GC is considered an important global health issue. The low survival rate of patients with GC is attributed to several factors, including the lack of understanding of GC pathogenesis and the lack of suitable prognostic biomarkers. Hence, identifying new molecular biomarkers will aid in GC diagnosis and treatment. Therefore, investigating the molecular mechanisms of GC is urgent and important.

GRKs phosphorylate G protein-coupled receptors to regulate their function, thereby affecting the downstream biological processes under the control of these receptors 13, 18-21. Previous studies have reported that GRK3 may act as an oncogene or a tumor suppressor gene in different cancers, depending on different tissues types, cancer types, and cancer stages. At present, the expression level of GRK3 in GC remains unknown. In this study, we observed that GRK3 expression in both GC tissues and cells was higher than that in normal gastric tissues and cells; GRK3 expression also showed a significant positive correlation with various clinicopathologic characteristics, such as lymphatic metastasis, distant metastasis, TNM stage, and vascular invasion. These results indicate that GRK3 acts as an oncogene in GC.

Although the correlation between GRK3 overexpression and prognosis has been reported in liver, colon, and pancreatic cancers, a consistent conclusion has not been obtained 14-16. To further examine the prognostic potential of GRK3, we analyzed the correlation between GRK3 expression and patient survival. From Kaplan-Meier survival curves, we observed that GC patients with high GRK3 expression had a significantly shorter disease-free survival and overall survival time than the patients with low GRK3 expression. This finding was identical to the findings in colon and pancreatic cancers. Multivariate Cox regression analysis also showed that the overexpression of GRK3 was an independent prognostic biomarker of GC. To the best of our knowledge, this is the first study reporting that GRK3 overexpression could be used as a biomarker for predicting patient prognosis in GC.

The results of previous studies on the effects of GRK on cell proliferation were inconsistent. Jiang et al. found that proliferation was inhibited in GRK3-knockdown RKO and LoVO cells, with identical results in xenograft experiments 15. Similar results were found in another study reporting that GRK3 is essential for the proliferation of SW620 colon cancer cells 22. However, a study on breast cancer reported no significant differences in proliferative capacity between GRK3-knockdown 66c14 breast cancer cells and cells in the control group 13. In the present study, we employed RNAi technology to examine the effects of GRK3 knockdown on cell proliferation in GC. GRK3 knockdown in HGC-27 cells significantly inhibited cell proliferation. Flow cytometry further revealed that GRK3 knockdown interfered with the transition from G0/G1 phase to S phase during cell cycle. This result was consistent with the results of two studies on colon cancer.

Most patients who died of GC are diagnosed when cancer cells have metastasized to other organs in the body. Further, cell invasion is closely associated with metastasis. With regards to studies on GRK3 and metastasis, Li et al. found that endothelial cell migration is significantly increased when GRK3 expression is increased. These researchers transplanted GRK3-knockdown cells into the prostate of SCID mice and found a significant decrease in tumor proliferation and metastasis. In addition, tumor microvessel density significantly increased when GRK3 was overexpressed in tumor cells, suggesting that GRK3 promoted angiogenesis. Consistent with the above conclusion, GRK3 expression was higher in metastatic tumors compared with tumors in the early stages 22. Our statistical analysis results revealed that GRK3 expression level was significantly correlated with lymphatic metastasis, distant metastasis, TNM stage, and vascular invasion. In this study, we also examined the effects of GRK3 knockdown on cell migration and invasion in GC. GRK3 knockdown inhibited HGC-27 cell migration and invasion, suggesting a correlation between GRK3 and GC.

There were some limitations to this study. We did not conduct an in-depth examination of the mechanism by which GRK3 promotes GC and signal transduction. Therefore, our future studies will focus on answering these questions.

At present, this is the first study to explore GRK3 expression in GC, the correlation of GRK3 overexpression with clinicopathologic characteristics and patient prognosis, and its function in proliferation, migration, and invasion in GC. Our findings showed that GRK3 overexpression can be used as a biomarker for predicting patient prognosis in GC and that GRK3 can be used as a target for the treatment of GC.

## Supplementary Material

Supplementary figures.

## Figures and Tables

**Table 1:** GRK3 expression in GC and its correlation with clinicopathologic characteristics

Variable	n	GRK3 expression		P*
		Score = 0 or 1 (n = 76)	Score = 2 or 3 (n = 68)	
**Age**				0.5888
≥ 66	92	47	45	
< 66	52	29	23	
**Gender**				0.7053
Male	93	48	45	
Female	51	28	23	
**Lauren classification**				0.7871
Intestinal	99	53	46	
Diffuse	45	23	22	
**Depth of tumor invasion**				0.1231
T1 + T2	36	23	13	
T3 + T4	108	53	55	
**Lymphatic metastasis**				0.0011
N0	47	34	13	
N1 + N2 + N3	97	42	55	
**Distant metastasis**				< 0.0001
Absent	126	76	49	
Present	18	0	18	
**TNM stage**				0.0035
I + II	65	43	22	
III + IV	79	33	46	
**Degree of differentiation**				0.1114
Poor	62	28	34	
Well to moderate	82	48	34	
**Vascular invasion**				0.0025
Absent	43	31	12	
Present	101	45	56	

*All statistical tests were 2-sided. Significance level: *P* < 0.05.

**Table 2:** Univariate and multivariate Cox regression analyses of prognostic biomarkers and survival in GC patients

Variable	Univariate		Multivariate	
	HR (95% CI)	P*	HR (95% CI)	P*
GRK3 Low expression vs. High expression	3.672 (2.127-6.338)	< 0.001	2.009 (1.076-3.753)	0.029
Age ≥ 66 vs. < 66	0.863 (0.520-1.432)	0.2568		
Gender Male vs. Female	0.774 (0.454-1.320)	0.348		
Lauren classification Intestinal vs. Diffuse	1.599 (0.958-2.667)	0.072		
Depth of tumor invasion T1 + T2 vs. T3 + T4	3.997 (1.718-9.302)	0.001	1.287 (0.479-3.460)	0.616
Lymphatic metastasis N0 vs. N1 + N2 + N3	7.109 (3.041-16.618)	< 0.001	1.985 (0.618-6.375)	0.250
Distant metastasis Absence vs. Presence	22.848 (9.059-57.631)	< 0.001	7.995 (3.137-20.372)	<0.001
TNM stage I + II vs. III + IV	6.672 (3.370-13.212)	< 0.001	2.320 (0.825-6.524)	0.111
Degree of differentiation Poor vs. Well to moderate	0.483 (0.292-0.800)	0.005	0.906 (0.522-1.571)	0.725
Vascular invasion Absent vs. Present	4.679 (2.120-10.327)	< 0.001	1.548 (0.635-3.770)	0.336

*All statistical tests were 2-sided. Significance level: *P*<0.05.

## Abbreviations

CI: confidence interval
GC: gastric cancer
GRK3: G protein-coupled receptor kinase 3
HR: hazard ratio
RIPA: radioimmunoprecipitation assay
RNAi: RNA interference
SDS-PAGE: sodium dodecyl sulfate polyacrylamide gel electrophoresis
shRNA: short hairpin RNA
