# Supplementary material for: GRK3 as a Prognosis Biomarker in Gastric Cancer
Source: J Cancer. 2022 Jan 31;13(4):1299–306. doi: 10.7150/jca.64748 (PMC8899382; doi:10.7150/jca.64748)
Supplement: Supplementary file 1 — Supplementary figures. [file jcav13p1299s1.pdf]

Supplementary figure legends

"GRK3 as a Prognosis Biomarker in Gastric Cancer" by Chia-Lang Fang, Yu-Feng Tian, Shiau-Shiuan Lin, Shih-Ting Hung, You-Cheng Hseu, Chun-Chao Chang, Chia-Lin Chou, Li-Chin Chen, Wen-Ching Wang, Kai-Yuan Lin, Ding-Ping Sun

Figure S1. The whole blot of GRK Western blotting in gastric tissues. In this blot, although other non-specific bands existed, GKR3 was the major band.

Figure S2. Verification of GRK3 knockdown in AGS cells, and the effect of stable GRK3 knockdown on cell growth, migration and invasion. The Western blotting results (A) indicate GRK3 was efficiently knockdown by shRNA treatment. (B) Stable GRK3 knockdown resulted in remarkedly decreased colony formation. (C) Stable GRK3 knockdown markedly decreased cell migration. (D) Stable GRK3 knockdown markedly decreased cell invasion.

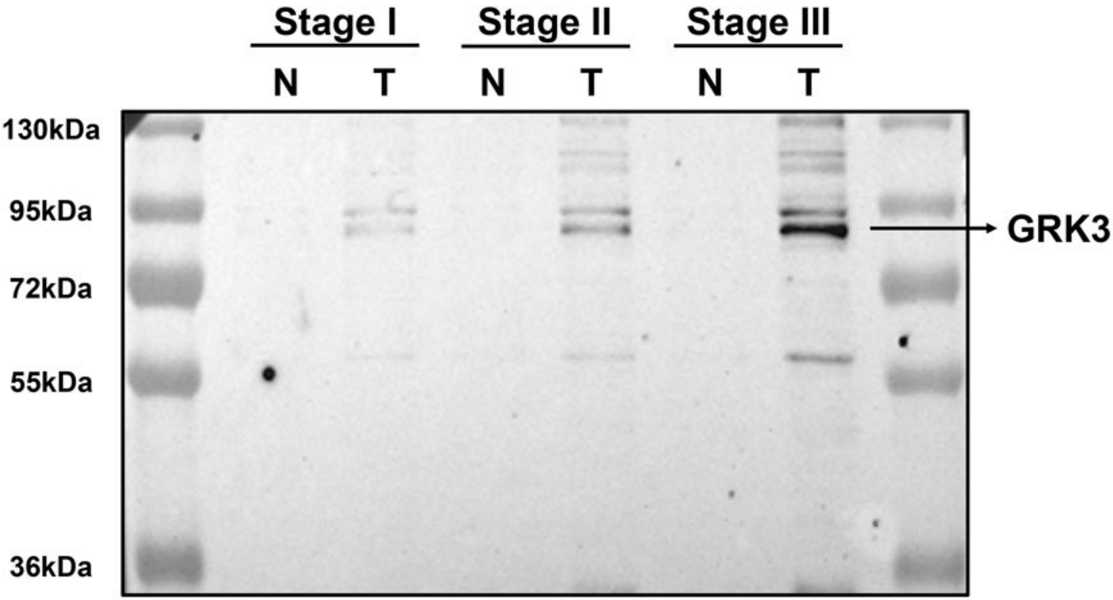
Stage I
Stage II
Stage III
N
T
N
T
N
T
130kDa
95kDa
72kDa
55kDa
36kDa
GRK3

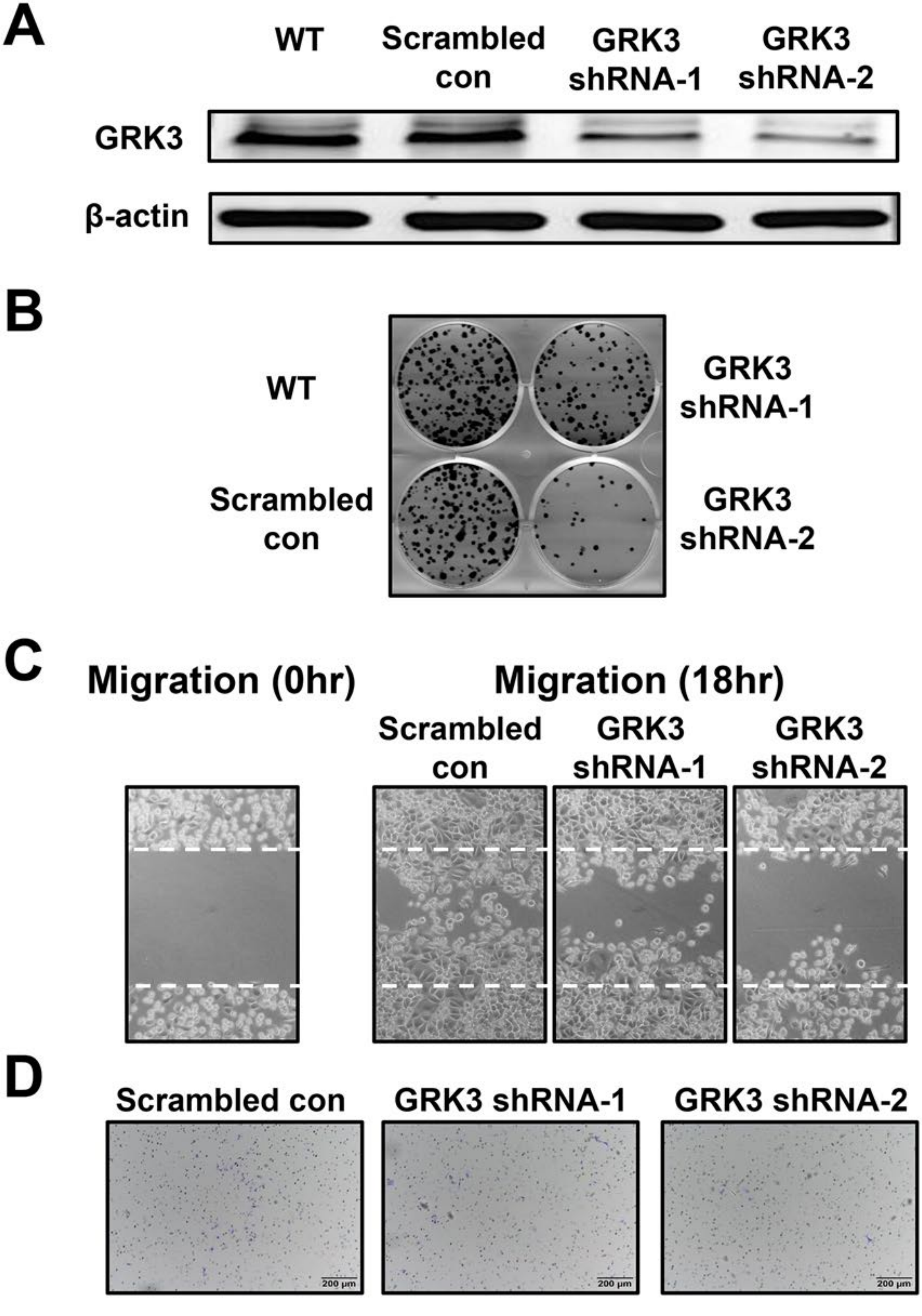

A
WT
Scrambled con
GRK3 shRNA-1
GRK3 shRNA-2
GRK3
β-actin
B
WT
GRK3 shRNA-1
Scrambled con
GRK3 shRNA-2
C
Migration (0hr)
Migration (18hr)
Scrambled con
GRK3 shRNA-1
GRK3 shRNA-2
D
Scrambled con
GRK3 shRNA-1
GRK3 shRNA-2
200 μm
200 μm
200 μm
